# Autism and Agricultural Pesticides: Integrating Data to Track Trends

**DOI:** 10.1289/ehp.115-a504a

**Published:** 2007-10

**Authors:** Victoria McGovern

The purpose of the CDC’s Environmental Public Health Tracking Program is to integrate diverse data sources for surveillance and research. In a demonstration project by program grantees, a powerful convergence of data on births, social services, and agriculture allows researchers to ask highly focused questions about the relationship between environmental exposures to farm pesticides and autism spectrum disorders (ASDs) in children **[*EHP* 115:1482–1489; Roberts et al.]**.

The study focused on ASDs in children whose mothers lived near well-defined sites of agricultural pesticide application in the California Central Valley, a 19-county swath spanning the Sacramento and San Joaquin River valleys. The team identified 465 children born in 1996–1998 who had received ASD-related diagnoses and services. They used state Department of Pesticide Regulation data to determine mothers’ residential proximity to pesticide applications at the time they gave birth. Data from 6,975 non-ASD children whose mothers had been pregnant in the same time and region served as controls.

The group set out to examine every combination of three factors: mother’s residential distance from the site of pesticide application, type of pesticide(s) applied, and stage of gestation at the time of pesticide use. Three time windows were of special interest: the period leading up to and covering central nervous system embryogenesis (1 week before conception through 7 weeks after), the period leading up to and covering neural tube development (4 days before conception through 24 days after) and overall gestation (2 weeks before conception through birth).

Because the number of possible combinations is high and the number of affected children relatively low, the study yielded only a preliminary view of how the three factors may interact. However one group of pesticides did stand out: organochlorines, including the commonly used dicofol and endosulfan, were associated with ASD out to a maternal residential distance of 1,750-meter from the site of application. Dicofol and endosulfan, which are used in the production of cotton, fruit, vegetables, beans, and nuts, account for 98% of the organochlorines applied in the Central Valley region.

Although the association between organochlorine exposure and ASDs points to a connection between the two, it does not indicate causality and does not consider other factors that may be involved. For the residences nearest to the organochlorine application sites (where the ASD association was the strongest), data around exposures came from only 8 cases and 105 controls. Of those, the focus is on the quarter who lived nearest sites where the greatest amounts of chemicals were applied. Still, the work, which used data routinely collected for other public uses, marks the need for further analysis of the relationship between organochlorines and ASDs, and lays the groundwork for asking difficult environmental health questions using available geographic, public health, and social services records.

## Figures and Tables

**Figure:**
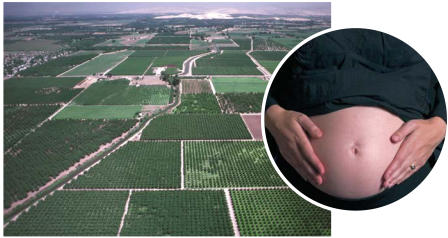
Data network Combining existing birth, social service, and chemical use data in new ways may reveal new environmental connections.

